# Reduction in trabecular meshwork stem cell content in donor eyes with primary open angle glaucoma

**DOI:** 10.1038/s41598-021-03345-1

**Published:** 2021-12-31

**Authors:** Yogapriya Sundaresan, Lakshmi Priya Manivannan, Shanthi Radhakrishnan, Krishnadas Subbiah Ramasamy, Muthukkaruppan Veerappan, Gowri Priya Chidambaranathan

**Affiliations:** 1 Department of Immunology and Stem Cell Biology, Aravind Medical Research Foundation, Madurai, 625020 Tamil Nadu India; 2 Department of Pathology, Aravind Eye Hospital and Post Graduate Institute of Ophthalmology, Madurai, 625020 Tamil Nadu India; 3 Glaucoma Clinic, Aravind Eye Hospital and Post Graduate Institute of Ophthalmology, Madurai, 625020 Tamil Nadu India

**Keywords:** Adult stem cells, Immunohistochemistry, Confocal microscopy

## Abstract

We previously identified and characterized human trabecular meshwork stem cells (TMSCs) based on high expression of ABCG2/p75 positivity and high nucleus to cytoplasmic ratio. These TMSCs expressing high ABCG2 and p75 were located to the insert region of the human TM. Additionally, we demonstrated an age-related reduction in the TMSC content which was significantly associated with TM cell loss. In continuation, this study was aimed to determine the TMSC content in glaucomatous donor eyes wherein a drastic reduction in TM cellularity has already been reported. Anterior segments from known glaucomatous (n = 6) and age-matched normal (n = 8) donors were dissected into four quadrants. A minimum of three sections from each quadrant were used for histopathological analysis as well as immunostaining. Analysis of hematoxylin and eosin-stained sections from glaucomatous tissues revealed a decrease in total TM cellularity, thickening of trabecular beams, fusion of trabeculae, absence of patent Schlemm’s canal compared to age-matched controls. In addition, the TM thickness at various positions of the meshwork and the coronal as well as the meridional diameters of the Schlemm’s canal were observed to be significantly reduced in glaucomatous eyes. Further, sections from both the groups were immunostained for universal stem cell marker ABCG2 and neural crest derived stem cell marker p75. The images were acquired using Leica SP8 confocal microscope. Quantification of total TM cellularity based on nuclear counterstain (mean ± SD) using ImageJ identified 69.33 ± 12.77 cells/section in control eyes. In glaucomatous donors, the TM cellularity was found to be reduced significantly to 41.83 ± 9.0 (p = 0.0007). In addition, a reduction in the percentage of TMSCs (cells with high ABCG2 expression and p75 positivity) was evident in glaucomatous donors (0.14 ± 0.17%) compared to age-matched controls (4.73 ± 5.46%) (p = 0.064). Thus, the present study confirmed the significant decline in TM cellularity and a reducing trend in the TMSC content, though this reduction was non-significant in glaucomatous donor eyes. Further studies are essential to elucidate the role of TMSCs in the pathogenesis of primary open angle glaucoma.

## Introduction

Glaucoma is the second leading cause of blindness^1,2^ affecting about 79.6 million people worldwide^3,4^. Among various types of glaucoma, primary open angle glaucoma (POAG) is the predominant form characterized by defective trabecular meshwork (TM) and by the loss of retinal ganglion cells leading to the degeneration of optic nerve^5^. The functional role of the human TM is to maintain the intraocular pressure (IOP) of the eye by regulating the aqueous humor (AH) outflow. The elevation in the IOP in turn affects the optic nerve through the mechanical changes at the lamina cribrosa leading to irreversible blindness^6^. Management of POAG has focused on the only treatable target, IOP^7,8^ and the treatment procedures comprise of incisional surgery, laser surgery and administration of medications such as prostaglandin analogs, beta blockers, alpha analogs, diuretics and cholinergic agonists. All the treatment modalities aim to relieve the IOP on the optic nerve either by reducing the rate of AH production or enhancing the aqueous outflow facility^9^.

Reports on the age-related changes in human TM demonstrated a significant reduction in total TM cellularity with increase in age^10–12^. This reduction in TM cell content is more prominent in patients with POAG where a gradient decline in TM cellularity was demonstrated in the filtering meshwork with inner tissues being more affected than outer tissues^13^. Thus, the need to analyse the stem cells in TM and their role in maintenance of tissue homeostasis is well indicated.

Adult tissue resident stem cells (SCs) are normally quiescent cells that divide when there is a need to maintain tissue homeostasis throughout life^14^. The presence of stem-like cells in TM was evident upon rapid cell proliferation in the region beneath Schwalbe’s line cells of the non-filtering TM following argon laser trabeculoplasty in human organ cultures^15^. Further, studies on primate and bovine eyes reported that these cells expressed putative SC markers and had label retention property^16,17^. We have earlier identified human TM stem cells (TMSCs) in isolated TM cells from native tissues based on two parameters—high ABCG2 (ATP-binding cassette G2 protein) expression and high N/C ratio. This method of identifying TMSCs was confirmed to be specific based on the co-expression of neural crest derived SC marker p75 and transition zone marker AnkyrinG. In addition, we confirmed that these TMSCs expressing high ABCG2 and p75 were restricted to the human TM insert region lying beneath the Schwalbe’s line. Further, analysis of isolated cells and sections of native TM in different age groups indicated a significant age-related reduction in TMSCs which correlated with the decrease in TM cells^12^. However, the status of the SCs in TM of human glaucomatous eyes remained unexplored where the TM cell reduction is known to be more pronounced^13^.

In addition to age-related decline, further reduction of adult SCs have been reported to be associated with disease pathogenesis^18,19^. Based on these reports we hypothesize that the severe decrease in TM cellularity in glaucomatous eyes is due to the reduction in the TMSCs. Understanding the status of TMSCs in glaucoma is significant particularly when the possibility of a cell-based therapy has been assessed in animal/human organ culture models^20–26^. Therefore, this study aimed to quantify the TMSC content based on the combined expression of ABCG2 and p75 in donor eyes with known history of POAG.

## Methods

### Sample collection

The donor eyes were obtained from Rotary Aravind International Eye Bank, Madurai, and the eye banks from Aravind Eye Hospitals at Tirunelveli and Coimbatore. The eyes were procured by the eye bank after obtaining the informed consent from the donor’s family. The human tissues were handled according to the tenets of the Declaration of Helsinki and the study was approved by the Institutional Ethics Committee of Aravind Eye Hospital, Madurai (IEC number: RES2016057BAS) and Institutional Committee for Stem Cell Research.

#### Glaucomatous globes

Glaucomatous globes from six donors with known history of POAG of age 58–92 years were included in the study (Table 1B). The glaucomatous globes were enucleated within 4 h after death and were fixed in 10% formalin within 10 h 20 min ± 2 h 40 min after the death of the donor. Complete clinical data was available only for two (S. No. 4 and 5 in Table 1B) of the four donors who underwent treatment in the host hospital from where the donor eyes were procured. For the other two donors (S. No. 1 and 6 in Table 1B), the history of POAG was obtained from the families but no additional details on the duration of the glaucoma or treatment could be obtained. As described earlier by Senthilkumari et al.^27^, the condition of POAG in these donors was confirmed by the pathologist [SR] after analysing the sagittal sections of optic nerve head (ONH) for retinal nerve fiber layer and lamina cribrosa thickness as well as ONH excavation (Supplementary Fig. 1).

**Table 1:** Demographic details of (A) control donor eyes and (B) glaucomatous donor eyes.

**(A)** Age matched control								
S. No.	Age (years)/sex	Eyes—paired or single	Duration of POAG (years) *	IOP during final visit (mm Hg)		Cup disc ratio		Cause of death
				Right eye	Left eye	Right eye	Left eye	
1	55/M	Single	Not POAG	NA	NA	NA	NA	Road traffic accident
2	56/F	Pair	Not POAG	NA	NA	NA	NA	Respiratory disease
3	83/F	Single	Not POAG	NA	NA	NA	NA	Cardiac arrest
4	87/M	Pair	Not POAG	NA	NA	NA	NA	Cardiac arrest
5	88/F	Single	Not POAG	NA	NA	NA	NA	Respiratory disease
6	90/M	Pair	Not POAG	NA	NA	NA	NA	Cardiac arrest
7	91/M	Single	Not POAG	NA	NA	NA	NA	Old age/natural death
8	93/F	Pair	Not POAG	NA	NA	NA	NA	Old age/natural death

**(B)** Glaucoma								
S. No.	Age (years)/sex	Eyes—paired or single	Duration of POAG (years)	IOP during final visit (mmHg)		Cup disc ratio		Cause of death
				Right eye	Left eye	Right eye	Left eye	
1	58/F	Pair	NA	NA	NA	NA	NA	Respiratory disease
2	80/F	Single	30	NA	NA	NA	NA	Respiratory distress
3	88/M	Single	20	NA	NA	NA	NA	Respiratory disease
4	88/M	Pair	23	22	44	0.9	1.0	Respiratory disease
5	90/F	Pair	22	16	16	1.0	0.9	Cardiac failure
6	92/F	Single	NA	NA	NA	NA	NA	Cardiac failure

(A) Details on the IOP and cup disc ratio of controls were not available. (B) Among the glaucomatous donor eyes, complete clinical data was available only for two (S.No. 4 and 5) of the four donors who underwent treatment in the host hospital from where the eyes were procured. For the other two donors (S. No. 1 and 6), the history of POAG was obtained from the families but no additional details on the duration of glaucoma or treatment was traceable. The pathology of glaucoma in these two donors was confirmed by histological analysis of posterior segments (Supplementary Fig.1). *These samples are from age-matched controls; NA – not available.

#### Age-matched controls

The whole globes not suitable for corneal transplantation were obtained from eight donors of age 55–93 years as age-matched controls (Table 1A). The inclusion criteria for the selection of age-matched donor tissues were (i) eyes enucleated within 4 h of death and received within 24 h for research, (ii) donors with no history of diabetes, ocular infection or systemic disease. Eyes from donors whose cause of death was due to poison or snake bite were excluded. The eyes were fixed by 17 h 25 min ± 5 h 30 min after death.

### Paraffin sectioning

The anterior segments of the control as well as glaucomatous eyes were dissected with intact iris/ciliary body and divided into four quadrants using a scalpel. Following fixation in 10% buffered formalin for 24 h, the quadrants were dehydrated through a series of graded ethanol and then infiltrated with paraffin wax in xylene. The tissues were then embedded into wax blocks and sectioned (5 μm). After deparaffinization, the sections were stained with hematoxylin and eosin (H and E) for histological analysis or antigen retrieval was carried out using 10 mM citrate buffer (pH 6.4) for 20 min at 90 °C followed by immunostaining^12^.

### Histological analysis

**Figure 1:**
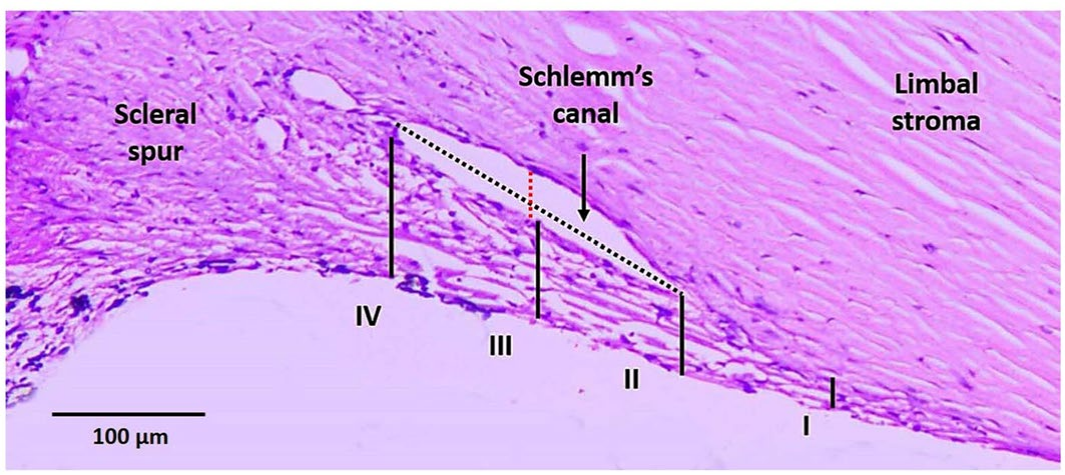
Representative light microscopic image of 90-year-old human TM control (S. No. 6, Table 1A) stained for H and E. The solid lines I to IV indicate the positions where the thickness of the TM was measured. The red and black dotted lines show the coronal and meridional diameter of Schlemm’s canal respectively.

All four quadrants from each eye (controls and glaucoma) and a minimum of three sections per quadrant were stained for H and E. As described by Dietlein et al*.*^28^, the thickness of the TM was measured at the following positions, (I) 100 µm anterior to the top of the Schlemm’s canal, (II) down the anterior end of the canal, (III) halfway down the canal and (IV) down the posterior end of the canal. In addition, the coronal and meridional diameter of the Schlemm’s canal was also measured as described by Yan et al*.*^29^(Fig. 1).

### Immunohistochemistry

The sections were blocked with avidin biotin blocking system (Thermofisher Scientific, Waltham, Massachusetts). Mouse monoclonal anti-BCRP antibody (anti-ATP Binding Cassette G2-Millipore, Billerica, MA) was added at a dilution of 1:20 in 5% BSA (Sigma Aldrich, St. Louis, Missouri). After overnight incubation at 25 °C, biotinylated secondary antibody (Goat anti-mouse IgG, DAKO-Glostrup, Denmark) was added at a dilution of 1:200 in 5% BSA and incubated for 1 h at 25 °C. Visualization was carried out using streptavidin-fluorescein isothiocyanate (FITC, BD Pharmingen, San Diego, CA) at a dilution of 1:1000 in 1 × PBS for 1 h at 25 °C. For double immunostaining, the second primary-rabbit anti-human p75 antibody (Promega, Madison, Wisconsin) was added at a dilution of 1:100. After overnight incubation, biotinylated secondary antibody (Mouse antirabbit IgG, Santa Cruz Biotechnology Inc, San Francisco, CA) was then added at a dilution of 1:200 and incubated for 1 h at 25 °C. Visualization of p75 staining was carried out using streptavidin Alexa Fluor 633 (Thermofisher Scientific, Waltham, Massachusetts) at a dilution of 1:500. Between the steps, the slides were washed with 1 × PBS. The stained sections were then mounted with Vectashield mounting medium containing propidium iodide (Vectashield, Burlingame, CA). Sections without adding primary antibody during immunostaining were used as a negative control.

### Confocal microscopy

Acquisition of confocal images was carried out using a laser scanning microscope (Leica SP8 confocal microscope, Germany) as previously described^12^. Briefly, fluorescent Z stack images were acquired with the following settings: the emission band width for FITC ranged from 496 to 535 nm using laser blue 488; for PI from 555 to 600 nm using laser green 552 nm and for Alexa Fluor 633 from 640 to 725 nm using laser red 633 nm. Using the above parameters, images were acquired from the insert region of the non-filtering TM to the posterior region of the meshwork where the TM attaches with ciliary body.

### Quantification of total TM cellularity and TMSC content

All four quadrants from each donor eye (controls and glaucomatous) and a minimum of three sections per quadrant were analysed. The area considered for quantification of total TM cells was based on Sundaresan et al.^12^. Briefly, the total TM cells were counted from the insert area of the non-filtering TM to the scleral spur and the ciliary body where the TM attaches posteriorly. The Schlemm’s canal endothelial cells and Schwalbe’s line cells were carefully excluded during quantification. The mean number of cells in the total TM were quantified based on PI staining. Cells double positive for both high ABCG2 and p75 (representing TMSCs) in each section were also counted and averaged. In addition, the percentage of cells immunopositive for these markers among the total number of nuclei in the TM was calculated^12^. The mean and SD of total TM cells and TMSC content in age-matched controls and glaucomatous donors were then calculated using the mean values from each donor.

### Statistical analysis

Statistical analyses were performed using Stata 14.0. The Shapiro–Wilk test was used to determine the normality. If the data had a normal distribution, an independent t-test was carried out. The Mann–Whitney *U* test was conducted if the data was skewed, a p-value of less than 0.05 was considered significant.

### Conference presentation

This manuscript was partly presented as a poster entitled “Quantification of Human Trabecular Meshwork Stem Cells in Glaucomatous Donors” at The Association for Research in Vision and Ophthalmology (ARVO), Vancouver, Canada, 2019 (Investigative Ophthalmology and Visual Science, *July 2019, Vol. 60*, *5152*).

## Results

### Histopathological changes of the TM in glaucomatous human eyes

**Figure 2:**
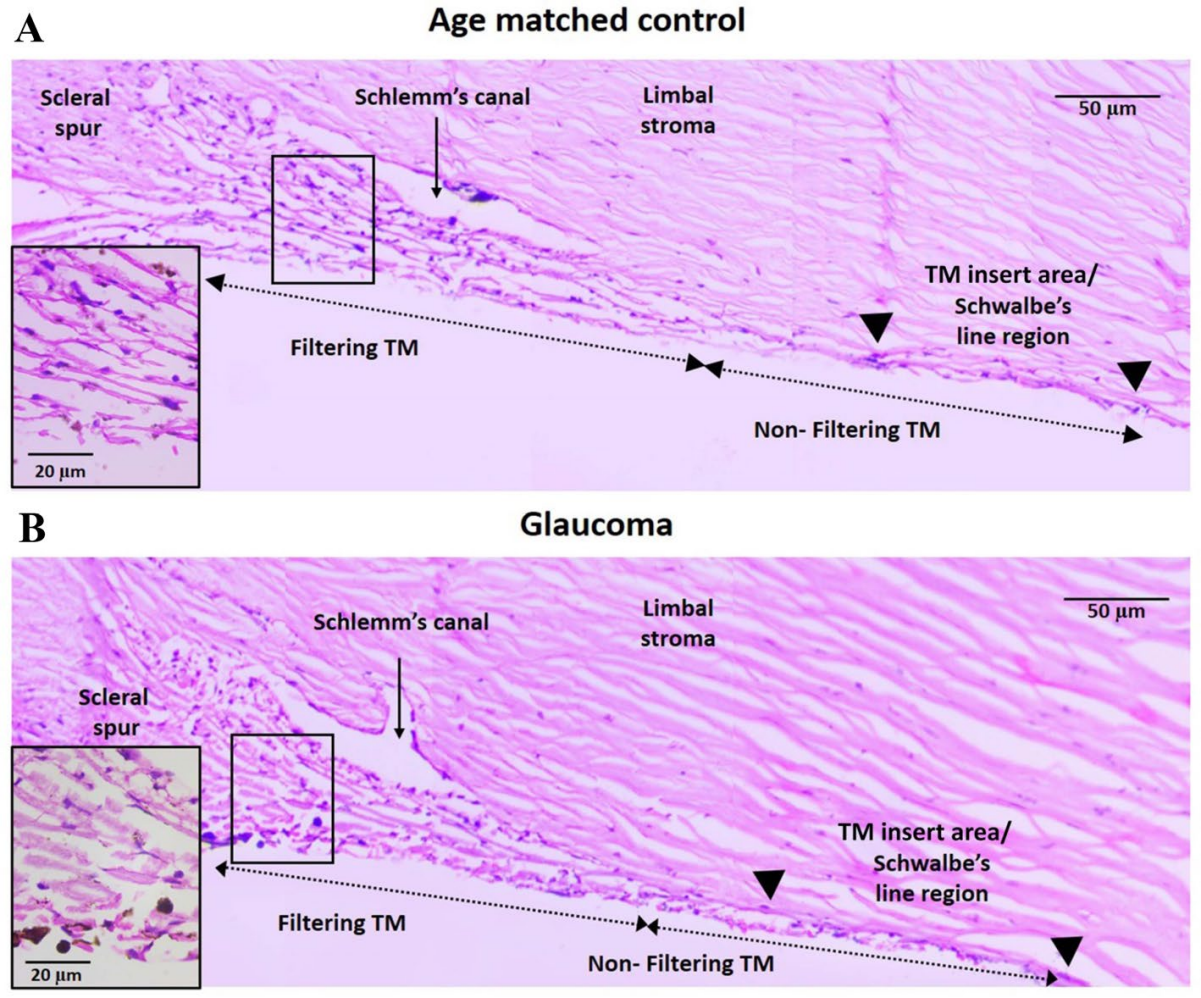
Montage of representative H and E-stained light microscopic images of TM section from 92-year-old glaucomatous donor (Table 1B, S. No. 6) and age-matched control (Table 1A, S. No. 8). Magnified image of the filtering region of the TM is shown in the inset. Reduced cellularity and thickening of trabecular beams were evident in glaucomatous donors compared to the controls.

In order to evaluate the anatomical changes in the glaucomatous TM, a minimum of three sections from all four anterior segment quadrants (of each eye) from six donors with known history of glaucoma and eight controls were analysed. H and E staining of TM from controls revealed abundant cellularity, slender trabecular beams and exhibited patent Schlemm’s canal (Fig. 2A). In contrast, the cellularity was reduced in all glaucomatous donor TM, thickening of trabecular beams was evident in four (S. No. 2, 4, 5 and 6, Table 1B), fusion of trabeculae, absence of patent Schlemm’s canal in three (S. No. 3, 4 and 5, Table 1B) and compressed meshwork in two (S. No. 3 and 4, Table 1B) (Fig. 2B).

**Figure 3:**
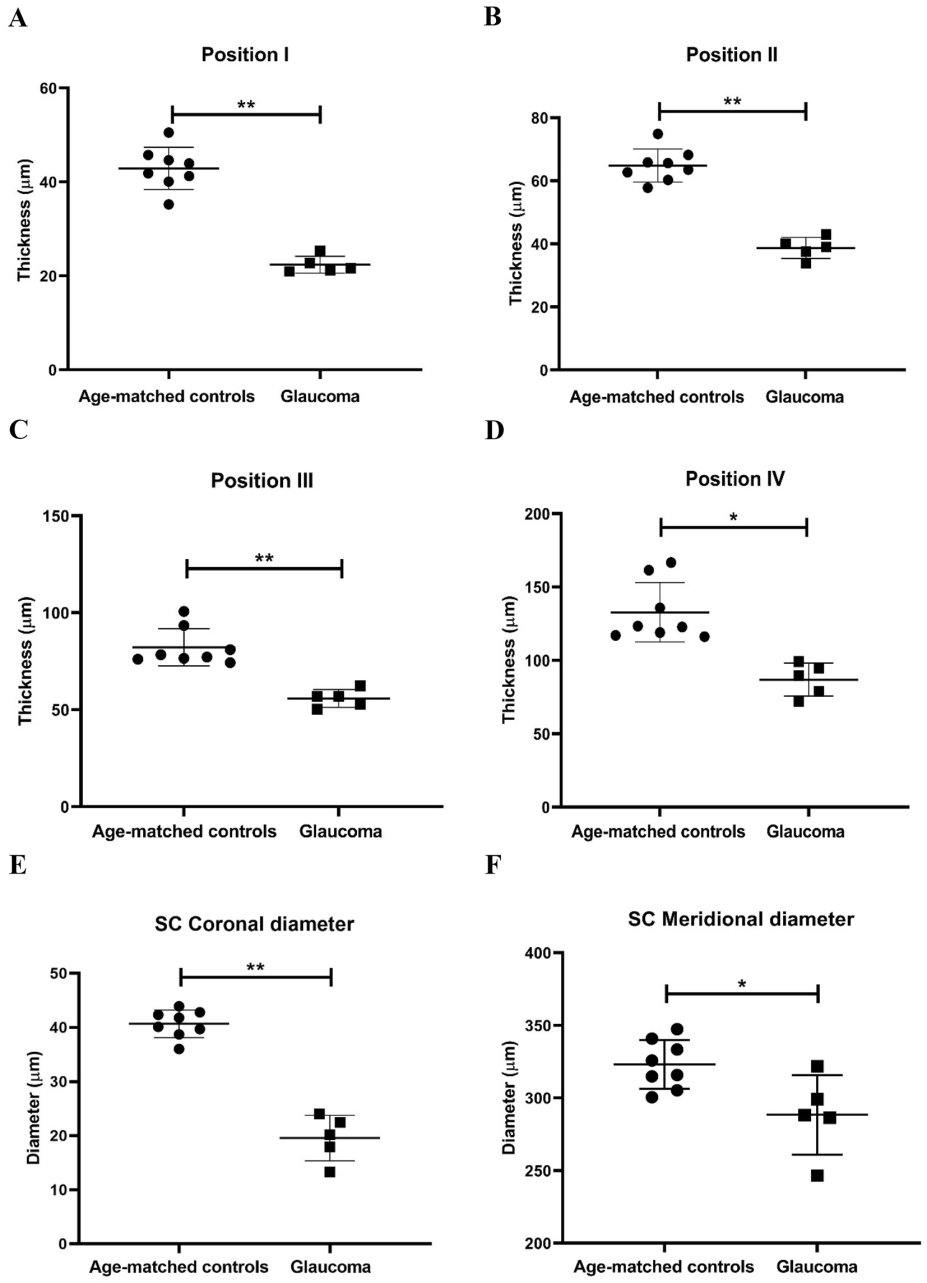
Scatter plots indicating the thickness of TM measured at Position I (**A**), Position II (**B**), Position III (**C**) and Position IV (**D**) in both controls and glaucomatous donors (positions as defined in Fig. 1). In addition, the Coronal (**E**) and Meridional (**F**) diameter of the Schlemm’s canal was measured. Significant reduction in TM thickness and coronal as well as meridional diameter was evident. Each dot represents the data from a single control donor and each square represents the data from a single glaucomatous donor (mean value of the measurement made in a minimum of three sections per quadrant). Symbols * and ** represents significant reduction (p < 0.05) and highly significant reduction (p < 0.0001) between the groups respectively.

Further, the thickness of the TM at position I, II, III and IV was measured to be 43.63 ± 5.05 µm, 65.60 ± 8.14 µm, 84.58 ± 14.77 µm and 136.5 ± 23.26 µm respectively in control tissues. This thickness significantly reduced to 22.45 ± 4.87 µm (p < 0.0001), 37.48 ± 8.91 µm (p < 0.0001), 56.32 ± 12.32 µm (p < 0.0001) and 87.78 ± 20.86 µm (p = 0.0002) in glaucomatous TM. In addition, the coronal and meridional diameter of the Schlemm’s canal was measured to be 40.61 ± 10.42 and 321.5 ± 54.6 respectively in age-matched controls that significantly decreased to 19.70 ± 4.95 (p < 0.0001) and 292.6 ± 28.78 (p = 0.02) in glaucomatous eyes (Fig. 3).

### Analysis of total TM cellularity in glaucomatous human eyes

**Figure 4:**
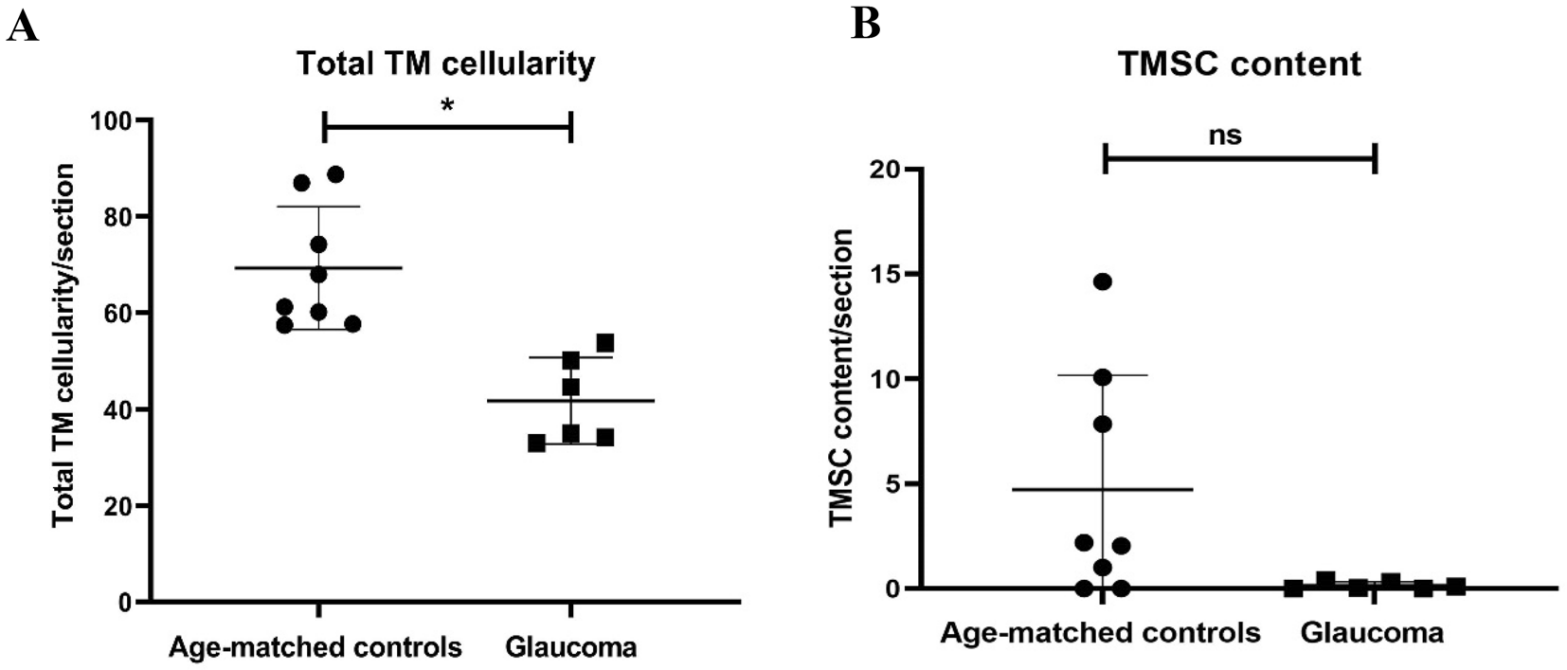
Scatter plots indicating the total TM cellularity (**A**), and percentage of TMSCs (**B**) in age-matched controls and glaucomatous TM. A significant (*) reduction in mean total TM cellularity (p = 0.0007) was observed in glaucomatous donors compared to controls. In addition, a decline in the percentage of TMSCs was also observed in glaucomatous eyes (p = 0.064). Each dot represents the data from a single control donor and each square represents the data from a single glaucomatous donor.

Analysis of total TM cellularity (mean ± SD) from all four quadrants of donor tissues based on the nuclear counterstain identified a significant reduction in glaucomatous donor eyes (41.83 ± 9.0 cells/section) compared to age-matched controls (69.33 ± 12.77 cells/section) (p value = 0.0007) (Fig. 4A, Table 2).

**Table 2:** Quantification of the mean total TM cell count and percentage of TMSCs in glaucomatous eyes and age-matched controls.

Donor tissue (age in years)	Mean total TM cell count/section	Mean percentage of TMSCs in total TM
**Age matched controls (n = 8)**		
55	87.0	10.09
56	88.8	7.86
83	68.00	2.20
87	57.46	0
88	74.25	1.01
90	60.17	14.63
91	61.25	2.04
93	57.72	0
Mean ± SD	69.33 ± 12.77	4.73 ± 5.46
**Glaucomatous donors (n = 6)**		
58	50.24	0.03
80	34.20	0
88	33.00	0
88	53.81	0.40
90	35.02	0.32
92	44.68	0.11
Mean ± SD	41.83 ± 9.0 (p = 0.0007)	0.14 ± 0.17 (p = 0.064)

Significant (*) reduction in mean total TM cellularity (p = 0.0007) was evident in glaucomatous donors compared to controls. A decrease in the percentage of TMSCs was observed between the groups albeit not significantly (p = 0.064).

### Total number of TMSCs in glaucomatous human donor eyes

**Figure 5:**
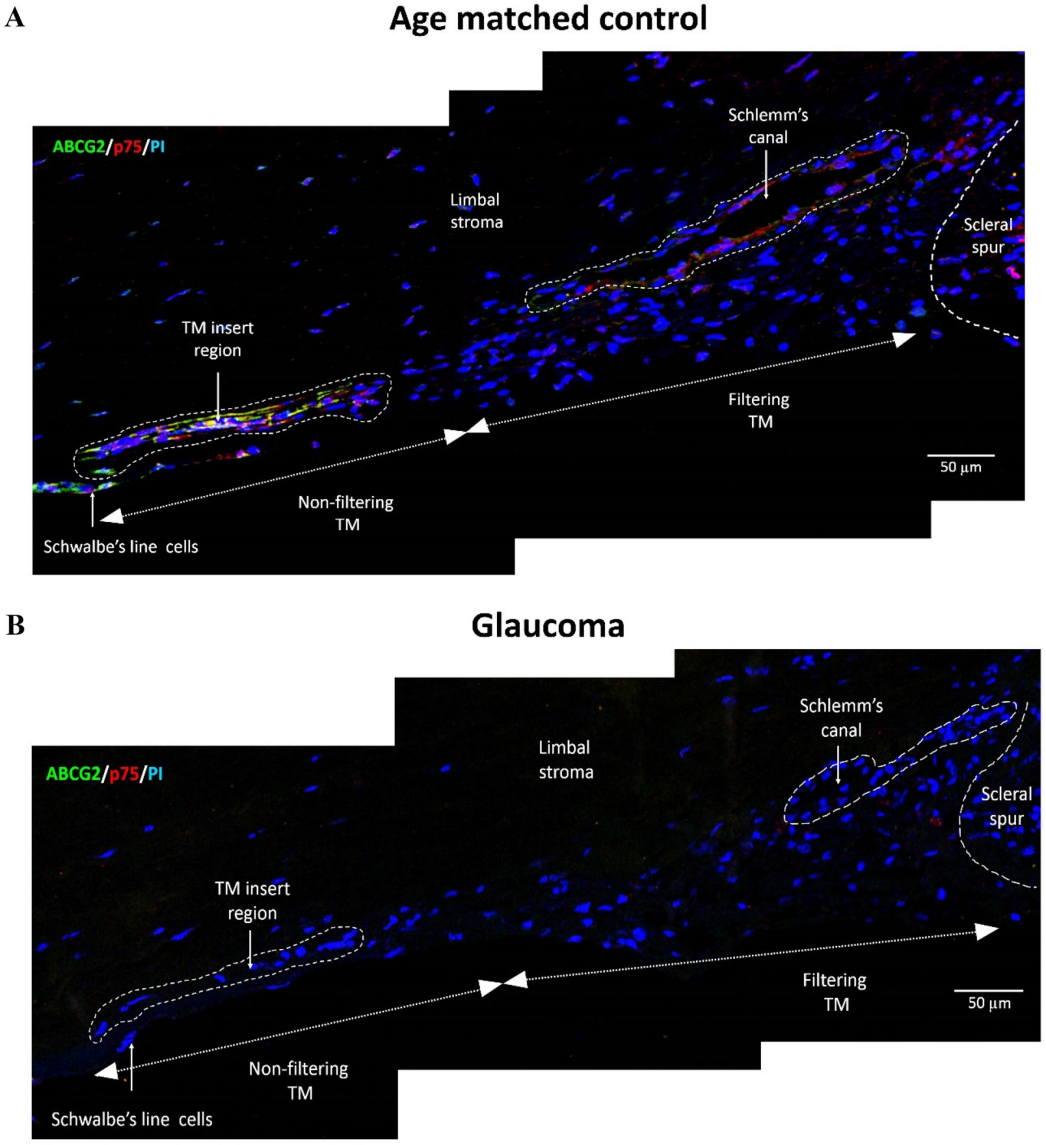
Montage of representative confocal images of TM from (**A**) age-matched control (Table 1A, S. No. 2, Age: 56 years) and (**B**) Glaucomatous donor (Table 1B, S. No. 1, Age: 58 years) double immunostained for ABCG2 (FITC-green) and p75 (Alexa 633-red), counterstained with PI (blue). The TMSCs identified as cells with high ABCG2 and p75 expression in the insert area were observed in the control TM. No ABCG2 or p75 expression was evident in the residual cells of the TM insert region in the glaucomatous donor.

Confocal microscopic analysis of TM sections from age-matched controls revealed that the expression of ABCG2 was higher in the TM insert region and Schwalbe’s line compared to the filtering region. But the expression of p75 was observed only in the TM insert region and Schwalbe’s line. Thus, TMSCs were identified as cells with high ABCG2 and p75 positivity in the insert region (4.73 ± 5.46%) of the TM (Figs. 4B, 5A, Table 2).

**Figure 6:**
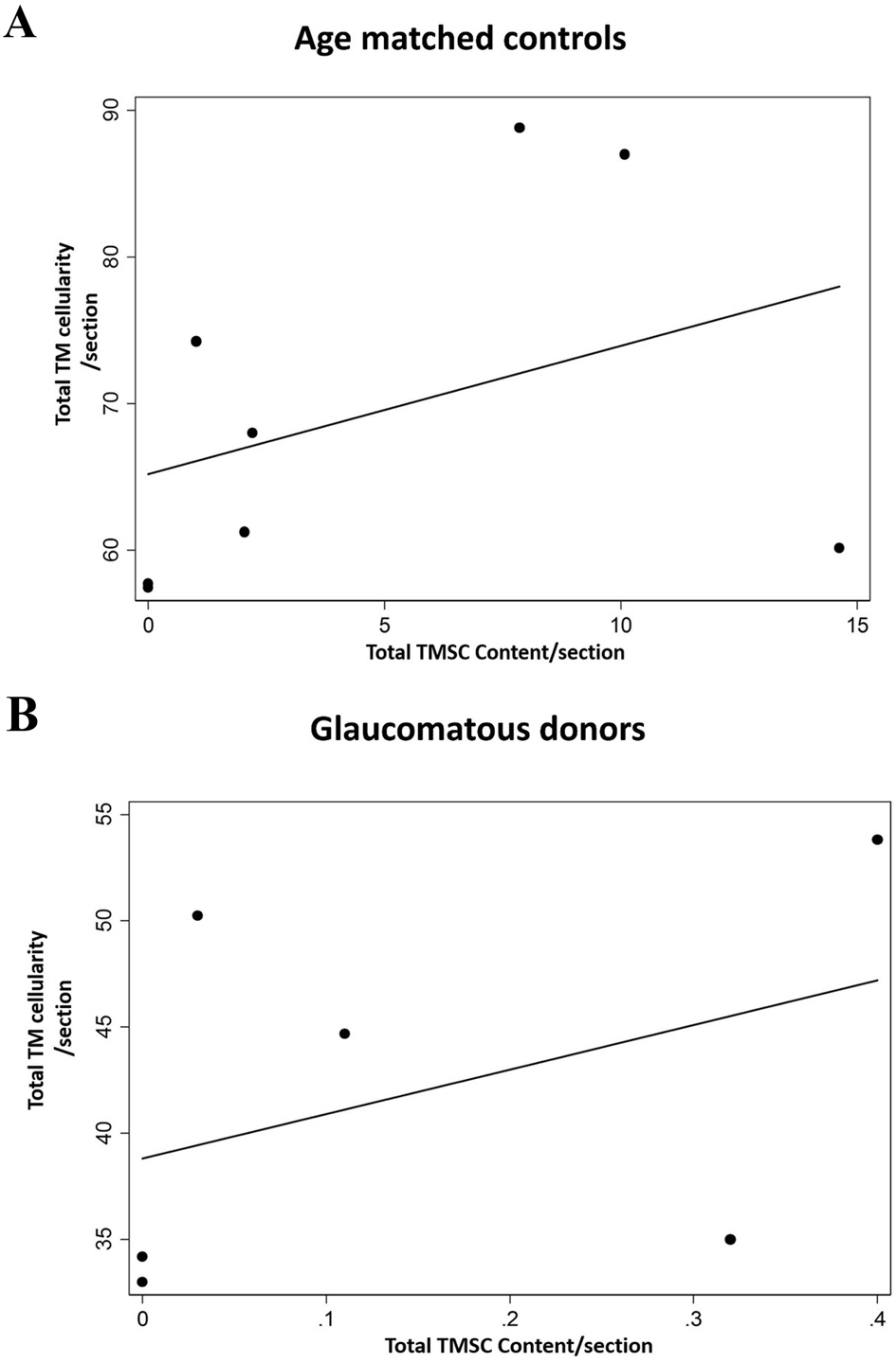
Correlation plot between total TM cellularity and TMSC content in age-matched controls and glaucomatous donor eyes. No significant correlation was observed between TM cellularity and TMSC content in (**A**) controls (p = 0.16) and (**B**) glaucomatous eyes (p = 0.071).

Further, the TMSCs reduced to 0.14 ± 0.17% (p = 0.064) in donor eyes with known history of POAG (Figs. 4B, 5B, Table 2), however this decrease was not significant. In addition, no significant correlation was observed between TM cellularity and TMSC content in controls (p = 0.16) and glaucomatous eyes (p = 0.071) (Fig. 6).

## Discussion

The presence of SCs in human TM that maintain tissue homeostasis was evident from the active proliferation of cells in the anterior, non-filtering region of the TM where it inserts into the cornea beneath Schwalbe's line following argon laser trabeculoplasty in human organ culture. The proliferating cells from beneath Schwalbe’s line migrate to the burn sites of the filtering meshwork to repopulate TM, thereby maintaining tissue homeostasis^15^. As reported in other adult tissue resident SCs^20,21,30^, any damage to TMSCs either a reduction in their content or functional loss might affect tissue regeneration. Our recent study on normal donor tissues demonstrated that the age-related reduction in TM cellularity to be significantly associated with decrease in TMSC content^12^. This was confirmed by the biphasic TMSC content among the age-matched controls in the present study. In addition to the age-related TM cell loss, further reduction in TM cell content has been reported in POAG which was associated with increased stiffness and resistance to AH outflow leading to elevated IOP^13^. Therefore, this study was carried out to analyse whether the drastic reduction in TM cellularity reported in POAG donors is associated with increased decline of TMSCs compared to age-matched controls.

In addition to reduced TM cellularity in glaucomatous eyes, other anatomical alterations in the TM that are associated with the development of POAG include thickening of trabecular beams and fusion of trabeculae due to extracellular matrix deposition^31,32^ along with a decrease in the number of giant vacuoles and pores of the inner wall of Schlemm’s canal endothelium^33,34^, shorter scleral spur^35^ as well as absence of patent Schlemm’s canal^36^. Histological observation of the TM sections from glaucomatous donors in this study indicated similar anatomical changes such as reduced cellularity, trabecular thickening and absence of patent Schlemm’s canal (Fig. 2). Further, a significant reduction in thickness of TM in various positions as well as the coronal and meridional diameters of Schlemm’s canal was found in glaucomatous donors (Fig. 3). These results were in agreement with the previous reports where the TM thickness and SC diameters were reduced in POAG compared to the controls^28,29^.

Quantification of total TM cellularity revealed a significant reduction in glaucomatous eyes compared to age-matched controls (Table 2), which confirmed the previous finding^13^. In order to elucidate whether the TM cell reduction in glaucoma is associated with the TMSC content, all the four quadrants of the TM tissues (both glaucomatous and age-matched donors) were analysed based on the higher expression of ABCG2 and p75 positivity^12^. The marker ABCG2 is a universal SC marker expressed in a wide variety of adult SCs. In addition, ABCG2 is also a molecular determinant of the side population phenotype of SCs that possess the efficient dye efflux mechanism owing to the higher expression of ABCG2^37^. We have earlier identified and quantified adult human limbal epithelial SCs^38^, buccal mucosal epithelial SCs^39^ and TMSCs^12^ based on the high ABCG2 expression in combination with high N/C ratio. Along with the expression of high ABCG2, p75, a neural crest derived SC marker was also used to identify the human TMSCs, since the human TM is neural crest in origin^40^. At present there is no direct proof that cells expressing high ABCG2 and p75 are the source of TM cells. Even though it is still possible on the basis of (i) Acott et al*.*^15^ wherein cells in the insert region were considered as SCs , (ii) our finding Sundaresan et al*.*^12^ that the cells expressing high ABCG2 and p75 to be located only in the TM insert region and (iii) our recent report Sundaresan et al*.*^41^, that the native TM-derived neurospheres expressing high ABCG2 and p75 differentiated to TM cells in vitro. Further studies are essential to establish that the cells expressing high ABCG2 and p75 maintain TM tissue homeostasis. Quantification of TMSC content in glaucomatous eyes based on the expression of these SC markers revealed a reduction compared to age-matched controls (Fig. 4, Table 2). However, this reduction had no significant correlation with the known TM cell loss. Exhaustion in adult SC content beyond that observed with ageing has been reported in coronary artery disease^30^, Duchenne muscular dystrophy^18^ and hypercholesterolemia^19^. Similarly, a decrease in TMSCs in donor eyes reported with glaucoma compared to age-matched controls might contribute to impaired tissue functioning, thereby increasing IOP.

To the best of our knowledge, this is the first study to assess the TMSC content in eyes from POAG donors where a reduction in the percentage of TMSCs in glaucomatous donors was observed. Besides, additional studies are required to understand the glaucoma TM pathobiology, specifically the functional status of the remaining TMSCs and their niche in these glaucomatous eyes. This basic research is essential for the development of a more appropriate SC- based therapy for patients with POAG.

## Supplementary Information

Supplementary Figure 1.
