# Supplementary material for: Reduction in trabecular meshwork stem cell content in donor eyes with primary open angle glaucoma
Source: Sci Rep. 2021 Dec 31;11:24518. doi: 10.1038/s41598-021-03345-1 (PMC8720087; doi:10.1038/s41598-021-03345-1)
Supplement: Supplementary file 1 — Supplementary Figure 1. [file 41598_2021_3345_MOESM1_ESM.docx]

**
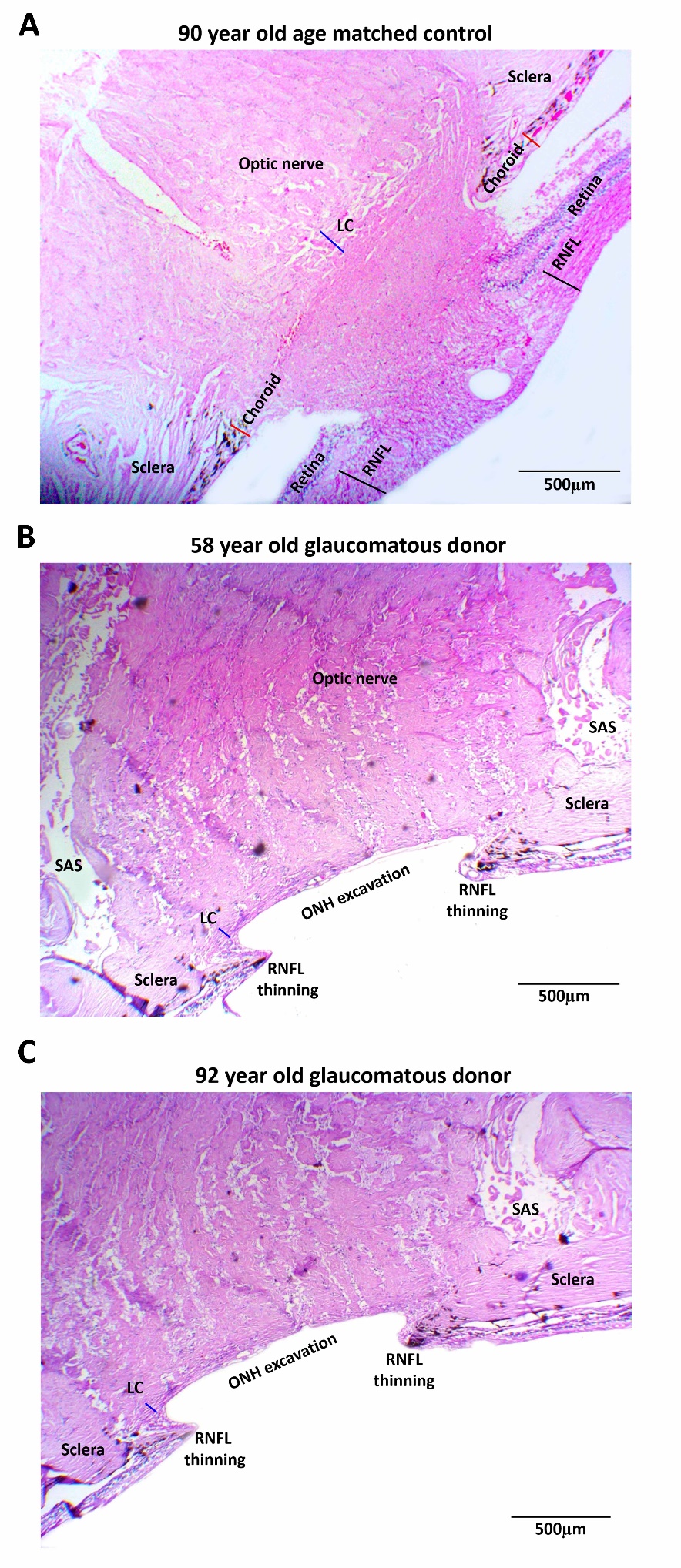
 Supplementary Figure**

**Supplementary Figure 1.** Representative light microscopic images of sagittal section of ONH of (A) 90-year-old age matched control (Table 1A, S. No 6), (B) 58-year-old glaucomatous donor (Table 1B, S. No 1) and 92-year-old glaucomatous donor (Table 1B, S. No 6) stained with hematoxylin and eosin. The median sagittal section indicated markedly reduced RNFL, severely compressed LC, marked excavation of ONH and axonal loss in glaucomatous donor eyes compared to controls. The blue vertical line marks the central thickness of LC and the black marks the RNFL thickness. RNFL-Retinal Nerve Fiber Layer; LC- Lamina Cribrosa; ONH- Optic Nerve Head; SAS-Sub Arachnoid Space.
